# Genome-wide association mapping for early maturity in kintoki bean (*Phaseolus vulgaris* L.)

**DOI:** 10.1270/jsbbs.24054

**Published:** 2025-03-27

**Authors:** Naoya Yamaguchi, Keisuke Tanaka, Kosuke Nakagawa, Hirokazu Sato, Akito Hosoi, Yozo Nakazawa

**Affiliations:** 1 Hokkaido Research Organization Central Agricultural Experiment Station, Higashi 6 sen Kita 15 gou, Naganuma-cho, Yubari-gun, Hokkaido 069-1395, Japan; 2 Tokyo University of Information Sciences, 4-1 Onaridai, Wakaba-ku, Chiba-shi, Chiba 265-8501, Japan; 3 Hokkaido Research Organization Tokachi Agricultural Experiment Station, 2, Minami 9 sen, Shinsei, Memuro-cho, Kasai-gun, Hokkaido 082-0081, Japan; 4 Hokkaido Research Organization Kitami Agricultural Experiment Station, Yayoi 52, Kunneppu-cho, Tokoro-gun, Hokkaido 099-1496, Japan; 5 Genome Research Center, Tokyo University of Agriculture, 1-1-1 Sakuragaoka, Setagaya-ku, Tokyo 156-8502, Japan; 6 Tokyo University of Agriculture, 196 Yasaka, Abashiri-shi, Hokkaido 099-2493, Japan

**Keywords:** common bean, maturity, flowering, genome-wide association study, marker-assisted selection

## Abstract

Japanese red or white common bean (*Phaseolus vulgaris* L.) cultivars, used to make sweetened boiled beans, are called “kintoki” beans. Kintoki beans are planted to precede winter wheat for crop rotation in Hokkaido, northern Japan. Therefore, early maturity is an important trait for them. The aim of this study was to map the genomic region associated with days to maturity in kintoki beans by genome-wide association study (GWAS). Significant single nucleotide polymorphisms associated with days to maturity were detected on chromosome 1 (Pv01) by GWAS in 3 years, and the candidate region for early maturity was mapped to a 473-kb region. Sequencing analysis indicated that *Phvul.001G221100*, a *phytochrome A3* gene, is likely to be responsible for early maturity in kintoki cultivars: the insertion of a cytosine in exon 1 at position 47 644 850 on Pv01 causes a frameshift that creates an early stop codon. Our findings suggest that the loss-of-function mutation of *Phvul.001G221100* is derived from a leading cultivar, ‘Taisho-Kintoki’, and is originated from a spontaneous mutation in the oldest kintoki cultivar, ‘Hon-Kintoki’. The DNA markers targeting the functional insertion of *phytochrome A3* will be useful for marker-assisted selection in kintoki bean breeding.

## Introduction

Common bean (*Phaseolus vulgaris* L.) is an important crop and is a major source of protein and essential nutrients. Japanese red or white bean cultivars used to make sweetened boiled beans are called “kintoki” beans. Hokaido, northern Japan, is a main kintoki beans production area (3930 ha in 2023), and accounts for more than 90% of the production of kintoki beans in Japan. It was in 1901, the breeding program of kintoki beans started in Hokkaido, and many kintoki bean cultivars have been released. Among them, ‘Taisho-Kintoki’ is a leading cultivar in Hokkaido. Its seed coat color is red-purple and it is preferred for processing (Koyama and Ushirogi 1957, Yamashita *et al.* 2014). Taisho-Kintoki began as an early-maturing plant in a miscellaneous ‘Hon-Kintoki’ population, the oldest kintoki cultivar planted in 1900’s to 1920’s, and was isolated by pure line breeding in 1957 (Koyama and Ushirogi 1957). Kintoki beans usually mature earlier than azuki beans and soybeans, and are planted to precede winter wheat for crop rotation in Hokkaido. Therefore, early maturity is an important trait for kintoki beans.

Maturity genes are known to be associated with floral regulation genes. For example, in soybean, time of flowering and maturity are controlled by *E* genes, which have various roles in maturity and photoperiod sensitivity (Miladinović *et al.* 2018). Genes *E3* and *E4* were isolated from soybean and characterized as *phytochrome A* genes (Liu *et al.* 2008, Watanabe *et al.* 2009). Phytochromes are well known as photoreceptors and affect the photoperiodic control of flowering (Johnson *et al.* 1994, Reed *et al.* 1994). Phytochrome A responds to very low fluence and high irradiance; other phytochromes respond to red/far-red–reversible low fluence (Whitelam *et al.* 1998). *E3* controls flowering under long-day conditions, and the recessive *e3* allele causes insensitivity to long daylength (Buzzell 1971). The effects of *E3* on days to flowering (DtF) and other traits in near-isogenic soybean lines were reported (Cober *et al.* 1996, Kawasaki *et al.* 2018, Yamada *et al.* 2012). In common bean, several classical genetic studies revealed that photoperiod insensitivity in Mesoamerican and Andean cultivars is conferred by recessive alleles at a major locus on chromosome 1, termed *Photoperiod* (*Ppd*; Wallace *et al.* 1993). *Phvul.001G221100*, a *phytochrome A3* gene and an ortholog of the soybean maturity gene *E3*, is a strong candidate for *Ppd* (Weller *et al.* 2019). The major loci controlling photoperiod sensitivity were also detected on chromosome 4 and 9, and nucleotide sequence analysis revealed the presence of polymorphisms between the parents of the mapping population for *CONSTANS-like 2* (*Phvul.004G046601*), *FLOWERING LOCUS D* (*Phvul.009G018700*), and *AGAMOUS-like 8* (*Phvul.009G203400*), as well as the ortholog to the soybean maturity gene *E1* (*Phvul.009G204600*) (González *et al.* 2016, 2021).

Genome-wide association studies (GWAS) can provide useful information in identifying loci associated with a particular trait. A reference genome for G19833 (Schmutz *et al.* 2014), an inbred landrace line of common bean derived from the Andean pool, supports the use of GWAS to identify associations between single nucleotide polymorphisms (SNPs) and phenotypic traits such as days to maturity (DtM) and DtF (Kamfwa *et al.* 2015, Keller *et al.* 2020, Moghaddam *et al.* 2016, Raggi *et al.* 2019), yield and yield components (Kamfwa *et al.* 2015, Keller *et al.* 2020, Moghaddam *et al.* 2016), morphological and color characters (García-Fernández *et al.* 2021), mineral content (Gunjača *et al.* 2021), cooking time (Diaz *et al.* 2021), and resistance to soybean cyst nematode (Shi *et al.* 2021). In particular, a significant SNP associated with DtM and DtF was located in the first intron of *Phvul.001G221100* in the population of Mesoamerican and Andean cultivars (Kamfwa *et al.* 2015).

The aims of this study were to identify significant SNPs associated with DtM in kintoki beans by GWAS, to search for the responsible genes by sequencing analysis, and to develop DNA markers linked to the genes for marker-assisted selection (MAS).

## Materials and Methods

### Plant materials

The 172 cultivars and breeding lines used in this study were grouped into red kintoki bean (*n* = 128), white kintoki bean (*n* = 27), red kidney bean (*n* = 13), and other (*n* = 4) (Supplemental Table 1). All 159 breeding lines were bred at the Tokachi Agricultural Experiment Station (TAES), Memuro, Hokkaido, and ranged in generation from F_6_ to F_12_. The following 10 cultivars were used for confirming a mutation and validation of SSR markers. ‘Taisho-Kintoki’ is a leading red kintoki cultivar in Hokkaido (Koyama and Ushirogi 1957, Yamashita *et al.* 2014). Four early-maturing cultivars are derived from it: ‘Fukura-Kintoki’ (Ebe *et al.* 2005a), ‘Akibare’, ‘Kita-Rosso’ (Saito *et al.* 2022), and ‘Tokei-B524’. ‘Kachidoki’ (Nakagawa *et al.* 2020), ‘Fukumasari’ (Sato *et al.* 1996), ‘Hokkai-Kintoki’ (Narikawa *et al.* 1980), ‘Fuku-Shirokintoki’ (Inuzuka *et al.* 1975), and ‘Fuku-Uzura’ (Ebe *et al.* 2005b) belong to the middle maturity group. Taisho-Kintoki, Fukura-Kintoki, Akibare, Tokei-B524, Kachidoki, Fukumasari, and Hokkai-Kintoki are red kintoki type; Fuku-Shirokintoki is white kintoki type; and Fuku-Uzura is Uzura type, with a pale tan seed coat with dark red-purple spots. All of the above cultivars are used for sweetened boiled beans, except Kita-Rosso, which is a red kidney type used for salad or soup. Two ‘Hon-Kintoki’ accessions, Hon-Kintoki-42001 and -42075, were collected by TAES in 1905 and 1982, respectively.

### Evaluation of days to maturity and flowering

The field test in 2021 was performed in Sarabetsu, a leased field owned by a farmer (42°66ʹN, 143°19ʹE). Those in 2022 and 2023 were performed at TAES (42°89ʹN, 140°07ʹE). Seeds were sown on 21 May 2021, 26 May 2022, and 25 May 2023. Each plot consisted of two or four 2.5-m rows spaced 60 cm apart, with 20 cm between hills and two plants per hill, at a plant density of 16.7 m^–2^. Each cultivar or line had one to five plots. The time of flowering was defined as when >50% of the plants in the plot were flowering. The time of maturity was defined as when >80% of the plants defoliated and turned yellow, with pods rattling when shaken. DtF and DtM were calculated from the date of sowing. DtM was evaluated in all 3 years and DtF in 2022 and 2023. Pearson’s correlation coefficient of DtM between years was determined.

### DNA extraction

DNA for ddRAD-seq and Illumina sequencing was extracted from young leaves by using a DNeasy Plant Mini Kit (Qiagen, Hilden, Germany) and NucleoBond HMW DNA (Takara, Japan), respectively. DNA for Sanger sequencing, and simple sequence repeat (SSR) and amplification refractory mutation system (ARMS) marker analysis was extracted from young leaves by a modified CTAB method (Suzuki *et al.* 2012).

### SNP detection by ddRAD-seq

Restriction-site-associated DNA (RAD) libraries were constructed for high-throughput DNA sequencing following the concept of the Flexible ddRAD-seq method (Ando *et al.* 2018). In brief, 100 ng of genomic DNA was fragmented by double digestion with *Eco*RI-HF and *Hin*dIII-HF (New England Biolabs, Ipswich, MA, USA). Fragmented DNA was ligated with custom fork adapters and size-selected using AMPure XP magnetic beads (Beckman Coulter, Brea, CA, USA). The custom adapter sequences were shown in Supplemental Table 2. After PCR amplification and mixing of each library in equal molar amounts, the RAD library mixture was sequenced by 2× 150-bp paired-end sequencing on a NextSeq 1000 sequencer (Illumina, San Diego, CA, USA). Raw reads containing adapters were trimmed in Trimmomatic v. 0.39 software (Bolger *et al.* 2014). The SNPs were profiled in ipyrad v. 0.9.84 software (Eaton and Overcast 2020). The *Phaseolus vulgaris* v. 2.1 reference genome sequence (https://phytozome-next.jgi.doe.gov/) was used for SNP mapping. The data have been deposited with links to BioProject accession number PRJDB18347 in the DNA Data Bank of Japan (DDBJ) BioProject database.

### Marker imputation and principal component analysis

Statistical analysis was conducted in R v. 4.2.2 software. As random forest imputation is useful for genotype imputation (Jarquín *et al.* 2014, Rutkoski *et al.* 2013), we performed marker imputation in the MissForest R package (Stekhoven and Bühlmann 2012). Principal component analysis (PCA) was conducted using the R prcomp function.

### Genome-wide association study for days to maturity and flowering

GWAS was performed in the rrBLUP R package (Endelman 2011), using the average values of DtM or DtF in each year. We used the P + K model (n.PC = 6) to control for population structure effects and relatedness. P and K indicate principal components and kinship, respectively. The R p.adjust function detected significant SNPs at a false discovery rate (FDR) of 0.05 (Benjamini and Hochberg 1995). Manhattan and Q–Q plots were created in the qqman R package (Turner 2018). Linkage disequilibrium (LD) blocks were estimated according to the Dʹ value by the method of Gabriel *et al.* (2002). LD analysis was performed in the Trio R package (Schwender *et al.* 2014).

### Illumina sequencing of Taisho-Kintoki

Genomic DNA of Taisho-Kintoki (1 μg) was sheared to an average size of 300 bp in an Adaptive Focused Acoustics sonicator (Covaris, Woburn, MA, USA). The genomic DNA library was constructed by using a Kapa Hyper Prep Kit (Kapa Biosystems, Wilmington, DE, USA). Fragmented DNA ligated with index adapters without PCR was size-selected in the 400–600-bp region by AMPure XP magnetic beads (Beckman Coulter). The DNA library mixture was sequenced by 2× 300-bp paired-end sequencing on a MiSeq sequencer (Illumina).

Raw read data were processed in CLC Genomics Workbench 23 software (Qiagen). After adapter trimming and quality filtering, the clean read data were mapped to the reference sequence in *P. vulgaris* v. 2.1. After local realignment of the mapped reads, duplicate PCR reads were discarded. Variant calling based on SNPs and indels was performed in the CLC Genomics Workbench “Fixed Ploidy Variant Detection” tool. The data have been deposited with links to BioProject accession number PRJDB18347 in the DDBJ BioProject database.

### Sanger sequencing

Sanger sequencing used forward primer Seq_m47644k (Table 1). PCR used Tks Gflex DNA polymerase (Takara, Japan) with an annealing temperature of 60 °C. After purification of the PCR product by ethanol precipitation, the sequencing reaction was conducted with a BigDye Terminator v. 3.1 Cycle Sequencing Kit (Thermo Fisher Scientific, Waltham, MA, USA). The DNA sequence was determined by ABI Prism 3500 Genetic Analyzer (Thermo Fisher Scientific). Sequence chromatograms were confirmed in the Poly Peak Parser R package (Hill *et al.* 2014).

### Validation of the genetic effects using SSR markers

PCR using primer sequences of five SSR markers (SSR_m47470k, SSR_m47528k, SSR_m47621k, SSR_m47782k, and SSR_m47913k; Table 1) used AmpliTaq Gold 360 DNA polymerase (Thermo Fisher Scientific) with an annealing temperature of 56 °C. The amplification products were analyzed on an ABI Prism 3500 Genetic Analyzer (Thermo Fisher Scientific) by GeneMapper software against a GeneScan-600 LIZ size standard as described (Suzuki *et al.* 2012, 2015). To validate the effects of the SSR markers, we used 29 breeding lines (F_5_ in 2023) derived from a cross between Kachidoki and Akibare for genotyping and phenotyping. For genotyping, the Akibare or Kachidoki alleles were determined with SSR_m47470k and SSR_m47913k. Lines with heterozygous or recombinant alleles were excluded. For phenotyping, the field test was conducted in 2023. Each plot consisted of a 2.5-m row (60 cm from neighboring rows), 15 cm between plants, at a plant density of 11.1 m^–2^. Student’s *t*-test was used to determine the significance of differences between genotypes.

### Development of ARMS marker and estimating the genetic effects

ARMS-PCR using primer sequences of ARMS_m47644k used Tks Gflex DNA polymerase with an annealing temperature of 60 °C (Table 1). The PCR products were analyzed using 2% agarose gel electrophoresis. A linear regression analysis was used to calculate the coefficients of determination (R^2^). The analysis was conducted using the R lm function. Estimated genetic effects were calculated as follows: (estimated genetic effects) = (average DtM with the Taisho-Kintoki allele) – (average DtM with the Kachidoki allele).

## Results

### SNP detection by ddRAD-seq and PCA

**Fig. 1.**
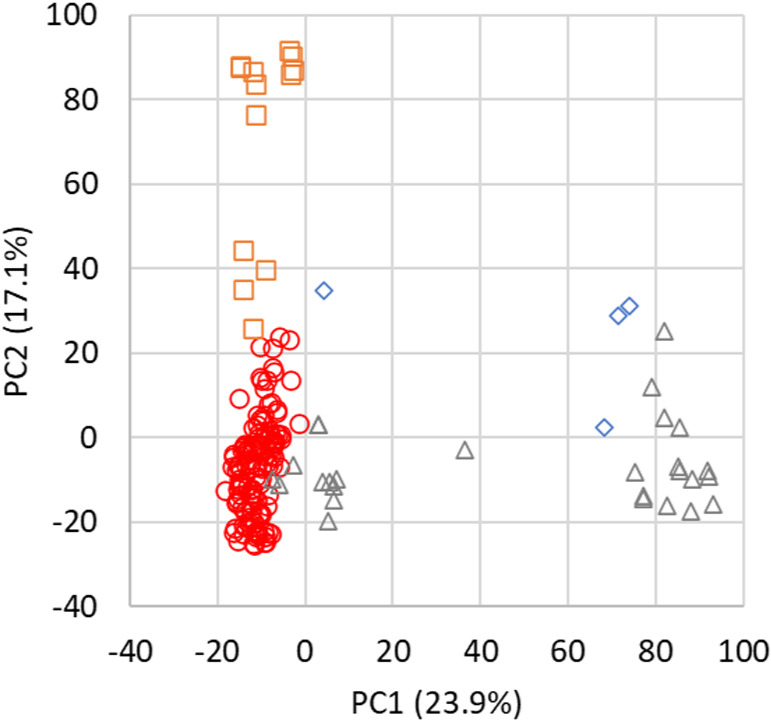
Principal component analysis (PCA) plots of PC1 against PC2 using 7339 SNP data. ○ Red kintoki bean. △ White kintoki bean. □ Red kidney bean. ◇ Other.

ddRAD-seq detected 41 748 genome-wide SNPs in the 172 cultivars and breeding lines after low-quality SNPs (missing >0.5) were removed. Missing data were imputed by random forest regression. After filtering for minor allele frequency (MAF >0.05), 7339 SNPs were retained. A plot of principal component 1 (PC1) against PC2 showed three clusters of red kintoki bean, white kintoki bean, and red kidney bean (Fig. 1). The sum of PC1 to PC6 explained 65.1%, enough to account for population structure. Thus, GWAS for DtM and DtF in each year was conducted using the P + K model (n.PC = 6) to control for population structure effects and relatedness.

### Genome-wide association study for days to maturity and flowering

**Fig. 2.**
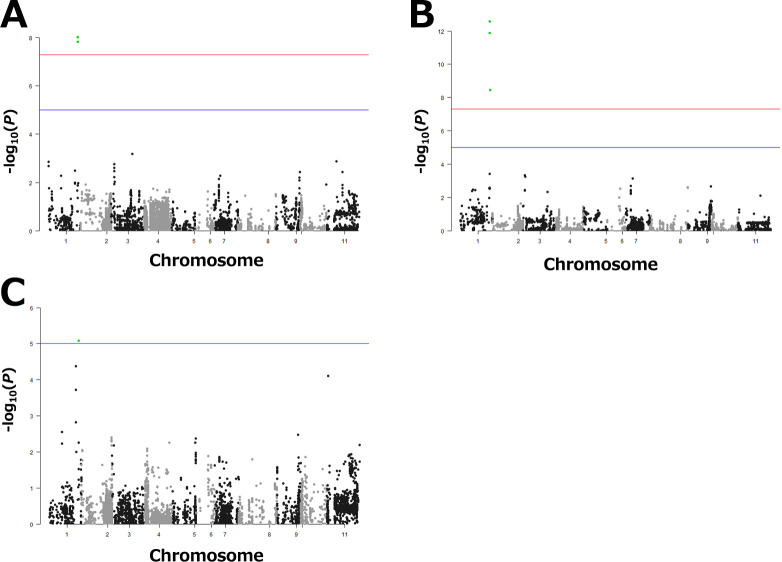
GWAS for days to maturity. Green dots are significant SNPs associated with maturity (FDR, *P* = 0.05). Red and blue horizontal lines mark 5 and 7.5 (–log_10_*P*), respectively. (A) Manhattan plot of phenotype data of 76 cultivars and breeding lines and 7242 SNP data in 2021. (B) Manhattan plot of phenotype data of 78 cultivars and breeding lines and 7294 SNP data in 2022. (C) Manhattan plot of phenotype data of 88 cultivars and breeding lines and 7302 SNP data in 2023.

**Fig. 3.**
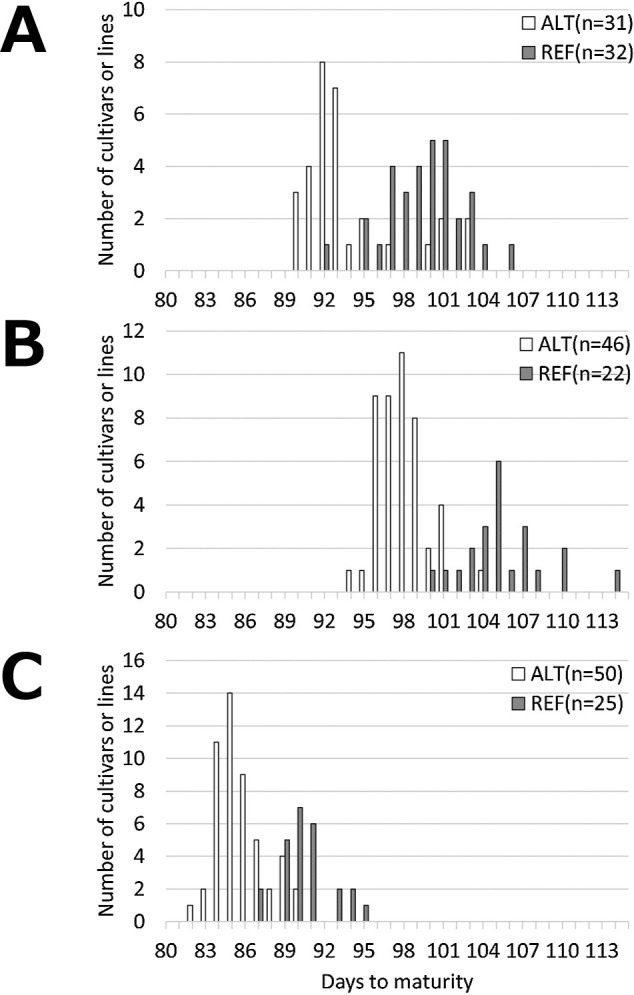
Frequency distributions of days to maturity (DtM) in each year. The genotypes at the SNP_47479438 locus are shown as (white) alternative (ALT, adenine) and (gray) reference (REF, thymine). (A) 2021: DtM, 90–106; range, 17 days. (B) 2022: DtM, 94–114; range, 21 days. (C) 2023: DtM, 82–95; range, 14 days.

There were significant positive correlations of DtM between years (Table 2). Highly positive correlations between DtM and DtF in 2022 (*r* = 0.820) and 2023 (*r* = 0.690) suggest that the gene responsible for early maturity is a floral regulatory gene. GWAS for DtM and DtF was conducted in each year (Fig. 2, Supplemental Fig. 1). Two or three significant SNPs associated with DtM were detected on chromosome 1 (Pv01) in each year (Table 3). Two significant SNPs associated with DtF were also detected on Pv01 in 2022, but none in 2023 (Table 3). DtM ranged from 90 to 106 in 2021 (Fig. 3A), from 94 to 114 in 2022 (Fig. 3B), and from 82 to 95 in 2023 (Fig. 3C). The phenotypic range is likely to affect GWAS scores.

GWAS suggested that SNP_47479438 and SNP_47479506 were tightly linked to a gene responsible for early maturity. No recombination occurred between them in the 172 cultivars and lines (Supplemental Table 1). The physical gap was found in the region between the SNP_47892313 and SNP_50630505 (Table 3), but this region included the 161 SNPs with a low MAF, not used for GWAS (Supplemental Table 3). Thus, we think this region may be the highly conserved in kintoki cultivars, and early maturity gene is unlikely to be located.

We found 16 LD blocks containing between 2 and 113 SNPs (total, 208 SNPs) on Pv01. SNP_47479438 and SNP_47479506 belong to LD block 14, indicating that the gene lies between positions 47 419 094 and 47 892 313 on Pv01 (Table 3, Supplemental Fig. 2). This candidate 473-kb region holds 53 protein-coding genes in Phytozome (*P. vulgaris* v. 2.1). This region includes *Phvul.001G221100* (47 642 032–47 647 745), a *phytochrome A3* gene, which is an ortholog of the soybean maturity gene *E3* (Watanabe *et al.* 2009) and a strong candidate for *Ppd* in common bean (Weller *et al.* 2019). *E3* affected DtF in soybean in many locations and DtM in one location (Yamada *et al.* 2012). The protein sequence similarity between *Phvul.001G221100* and *E3* is 94.7% in Phytozome. The other 52 genes are not associated with maturity or floral regulation, according to gene annotation. Therefore, we focused on *Phvul.001G221100* as a candidate gene for early maturity in kintoki cultivars.

### Sequencing of the candidate 473-kb region in Taisho-Kintoki

**Fig. 4.**
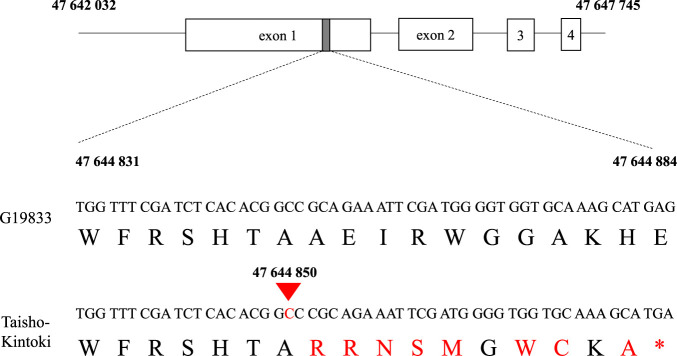
Insertion mutation in exon 1 of *Phvul.001G221100*. An insertion of C at position 47 644 850 was found in the Taisho-Kintoki sequence. Large letters indicate amino acid sequences; * = stop codon.

Whole-genome resequencing of Taisho-Kintoki resulted in a total of ~13.6 Gb. The total contig length of the reference genome G19833 is reported as 531 Mb in Phytozome (*P. vulgaris* v. 2.1), and thus the genome sequence of Taisho-Kintoki has ~25.6× sequence coverage. Comparative analysis between G19833 and Taisho-Kintoki revealed 270 mutations in the candidate 473-kb region, and two non-synonymous SNPs were found in *Phvul.001G221100* and *Phvul.001G223000* (Supplemental Table 4). Taisho-Kintoki had an insertion of a C at position 47 644 850 in exon 1 of *Phvul.001G221100* in all 11 reads, and this frameshift created a stop codon 11 codons downstream (Fig. 4). We defined this insertion as a loss-of-function mutation of *Phvul.001G221100*.

A non-synonymous SNP was also found in *Phvul.001G223000* (Supplemental Table 4). This gene codes protein transport protein *SEC24* in Phytozome. Sanger sequencing in the proximal region of exon 1 of *Phvul.001G223000* identified a non-synonymous SNP at position 47 811 067 in both Hon-Kintoki and Taisho-Kintoki (Supplemental Fig. 3). Thus, this SNP is unlikely to be associated with early maturity in Taisho-Kintoki.

### Confirming the insertion mutation in exon 1 of *Phvul.001G221100*

**Fig. 5.**
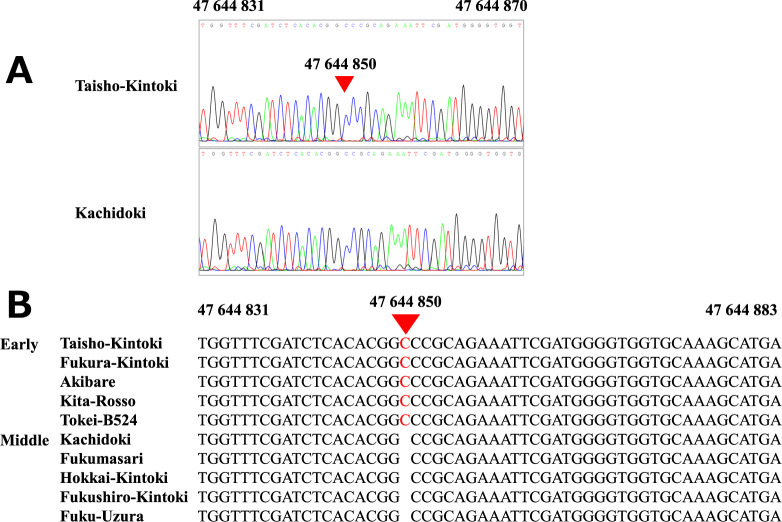
Confirming an insertion mutation in exon 1 of *Phvul.001G221100* by Sanger sequencing. (A) Representative sequence chromatograms of Taisho-Kintoki (early) and Kachidoki (middle). (B) Comparison of sequences of the 10 cultivars.

Sanger sequencing of ~300-nt sequences in the proximal region of exon 1 of *Phvul.001G221100* in ten cultivars (Fig. 5A) identified an insertion of a C at position 47 644 850 in all five cultivars in the early maturity group but in none of the five cultivars in the middle maturity group (Fig. 5B). These results indicate that the loss-of-function mutation due to the C insertion is likely to be important for early maturity.

### Validation of the effect of the SSR markers tightly linked to *Phvul.001G221100*

**Fig. 6.**
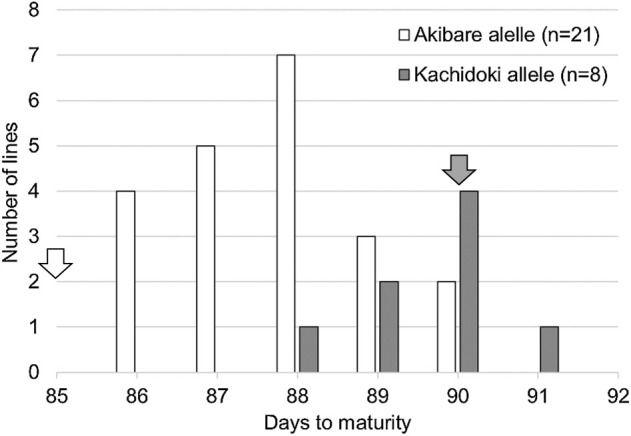
Frequency distribution of days to maturity in 29 breeding lines derived from Kachidoki (middle; average, 89.6 days) × Akibare (early; average, 87.7 days; *P* < 0.001) in 2023. Marker genotypes were determined with markers SSR_m47470k and SSR_m47913k. Arrows indicate parental values.

We validated the effects of five SSR markers (SSR_m47470k, SSR_m47528k, SSR_m47621k, SSR_m47782k, and SSR_m47913k; Table 1) in the same ten cultivars. All five markers were polymorphic between early and middle maturity groups, and the marker genotypes were the same in the early maturity group (Table 4). We also validated the effects of the markers SSR_m47470k and SSR_m47913k in 29 breeding lines derived from Kachidoki (middle) × Akibare (early). The average was significantly shorter in the lines with the Akibare alleles than in those with the Kachidoki alleles (*P* < 0.001, Fig. 6). These results indicate that these SSR markers, tightly linked to *Phvul.001G221100*, will be useful for MAS in kintoki bean breeding.

### Estimating the genetic effects using ARMS marker

**Fig. 7.**
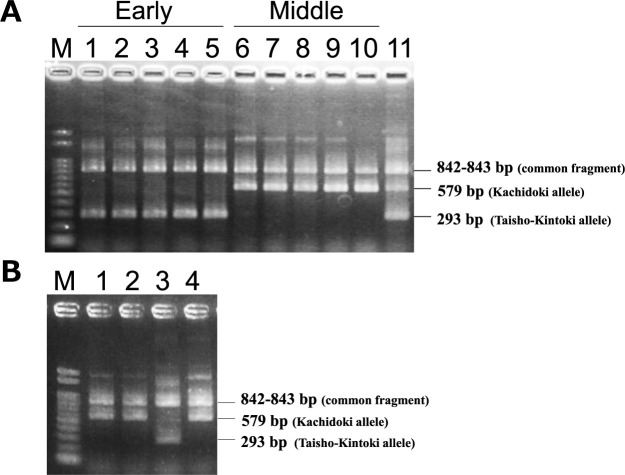
Agarose gel electrophoresis of ARMS-PCR products. (A) M, 100 bp DNA ladder marker; Lane 1, Taisho-Kintoki; Lane 2, Fukura-Kintoki; Lane 3, Akibare; Lane 4, Kita-Rosso; Lane 5, Tokei-B524; Lane 6, Kachidoki; Lane 7, Fukumasari; Lane 8, Hokkai-Kintoki; Lane 9, Fuku-Shirokintoki; Lane 10, Fuku-Uzura; Lane 11, F_1_ plant from a cross between Akibare and Kachidoki. (B) M, 100 bp DNA ladder marker; Lane 1, Hon-Kintoki-42001; Lane 2, Hon-Kintoki-42075; Lane 3, Taisho-Kintoki; Lane 4, Kachidoki.

We developed the ARMS marker identifying an insertion of a C at position 47 644 850 (Table 1). The ten cultivars were genotyped using the ARMS marker, and the results were identical to those of Sanger sequencing (Fig. 7A). We named the allele with an insertion of a C at position 47 644 850 (ALT) as the Taisho-Kintoki allele, and the reference allele (REF) as the Kachidoki allele. The 172 cultivars and breeding lines were genotyped using this marker (Supplemental Table 1), and the effects of this gene were estimated by a linear regression analysis (Table 5). The genetic effects on maturity were estimated to be from 4.8 to 7.3 days, and coefficients of determination for the difference in alleles were from 40.5 to 66.3% in each year. These results indicate that this gene will be highly effective.

### Estimating the origin of the insertion mutation

To estimate the origin of an insertion of a C at position 47 644 850, two Hon-Kintoki accessions were genotyped using the ARMS marker (Fig. 7B). Both accessions had the Kachidoki alleles, indicating that this insertion is originated from a spontaneous mutation in Hon-Kintoki.

## Discussion

Early maturity in kintoki cultivars is an important trait for crop rotation. We detected significant SNPs associated with DtM on Pv01 by GWAS in 3 years, and mapped the candidate region for early maturity to a 473-kb region (Fig. 2, Table 3). Sequencing analysis suggested that *Phvul.001G221100*, a *phytochrome A* gene, is responsible for early maturity in kintoki cultivars, and that a C insertion causing a loss-of-function mutation is important (Figs. 4, 5, Table 4). The four early-maturing cultivars—Fukura-Kintoki, Akibare, Kita-Rosso, and Tokei-B524—had the identical variant (Fig. 5) and the same five SSR marker genotypes as in Taisho-Kintoki (Table 4), and have Taisho-Kintoki in their pedigrees. Taisho-Kintoki began as an early-maturing plant in a miscellaneous Hon-Kintoki population in 1935 and was isolated by pure line breeding in 1957 (Koyama and Ushirogi 1957). In this study, two Hon-Kintoki accessions had no insertions at position 47 644 850 (Fig. 7B). Therefore, the loss-of-function mutation of *Phvul.001G221100* in Taisho-Kintoki originated from a spontaneous mutation in Hon-Kintoki, and new early-maturing cultivars such as Akibare (released in 2019) are likely to have inherited the mutation based on selection for the early maturity phenotype.

There are two prior reports of significant SNPs associated with DtM or DtF detected in the proximal region of *Phvul.001G221100*. Kamfwa *et al.* (2015) reported a significant SNP in the first intron of *Phvul.001G221100* in a population of Mesoamerican and Andean cultivars, and suggested *Phvul.001G221100* as a candidate gene for early maturity. As kintoki cultivars account for 89.5% in the population used in this study (Supplemental Table 1), the population structure in this study may be different from that in Kamfwa *et al.* (2015). Weller *et al.* (2019) sequenced *Phvul.001G221100* in a population of Mesoamerican and Andean cultivars; they found 61 polymorphic sites across the *Phvul.001G221100* coding sequence and defined 23 haplotypes. The Andean domesticated cultivar ‘Midas’ had a C insertion at position 47 644 850, identical to that in Taisho-Kintoki (Fig. 4; Weller *et al.* 2019). Midas is a snap bean cultivar with white seeds, and the pedigree of Midas is not available. Further studies will be needed to explain why Midas and Taisho-Kintoki have the identical variant despite their different origins.

Liu *et al.* (2008) found multiple homologs of the *phytochrome A* gene in the soybean genome and reported *E4* as a paralog of *E3*. Interestingly, there are multiple homologs of *phytochrome A* in the common bean genome (*P. vulgaris* v. 2.1). *Phvul.007G206800* on Pv07 is a *phytochrome A* gene; its protein sequence similarity to soybean *E4* is 97.8%, and thus *Phvul.007G206800* is likely to be an ortholog of *E4*. No significant SNPs were associated with DtM and DtF on Pv07 in the population used here, but *Phvul.007G206800* could affect maturity in other populations. To clarify the mechanisms of phytochromes in common bean, further studies of the functions of *Phvul.007G206800* are required.

DtM and DtF are affected by climatic and environmental conditions. 2023 was warmer than 2021 and 2022. In such conditions, the phenotypic range of maturity narrows in kintoki cultivars, and it is hard to evaluate maturity precisely; for example, that of DtM in the Kachidoki × Akibare lines was only 6 days in 2023 (Fig. 6), and that in the GWAS population was 14 days in 2023, smaller than in 2021 and 2022 (Fig. 3). We believe that MAS using the ARMS marker identifying the loss-of-function mutation of *Phvul.001G221100* (Fig. 7, Table 1), which is unaffected by climatic and environmental conditions, will prove effective for improving maturity.

In summary, we identified significant SNPs associated with DtM and DtF on Pv01. *Phvul.001G221100*, a *phytochrome A3* gene, is likely to be responsible for early maturity in kintoki cultivars, and the SSR and ARMS markers linked to it will be useful for MAS in kintoki bean breeding.

## Author Contribution Statement

NY, KT, and YN designed the research. KT and AH conducted the ddRAD-seq and resequence experiments. NY conducted the SSR marker and Sanger sequence experiments. NY, KN, and HS conducted the field experiments. NY, KT, KN, HS, AH, and YN analyzed the data. NY and KT wrote the manuscript. All authors read and approved the manuscript.

## Supplementary Material

Supplemental Figures

Supplemental Table 1

Supplemental Table 2

Supplemental Table 3

Supplemental Table 4

## Figures and Tables

**Table 1.** Primer sequences used in this study

Marker	Start position (forward primer)	Forward primer	Reverse primer
SSR_m47470k	47 469 891	TGAACATTGGTCTTCCGTTA	AATCTGCCATTGGATTGTTT
SSR_m47528k	47 528 421	TCTTGTATACCAAACTCTAAGTAA	TAGCTACACGATTAAATGGTATT
SSR_m47621k	47 621 111	ACTTGTGAAGTGTTGAATGGT	ACCTAACTCGTTGTGAACAC
SSR_m47782k	47 782 026	AAGTAATGAGTCACGTATATTTCAATC	TATAACTATTATGAGTTGTAGGTGTC
SSR_m47913k	47 913 321	AGAAATGTTATTCAAAGAGCTATA	TGCAAGTTGTTATTCATGCTA
Seq_m47644k	47 644 763	CTGCTACTCTTGGTGATATAACT	GCATATGTGCTTATCTCCA
ARMS_m47644k*^a^*	47 644 285	GTTATGGCGGTTGTAGTCAATG (outer)	TCGAATTTCTGCGGCTGT (Kachidoki allele-specific)
	47 644 836	TCGATCTCACACGGCACG (Taisho-Kintoki allele-specific)	AGTCACTGCTTCCAGATCCTG (outer)

*^a^* ARMS-PCR was conducted using four primers. Underlined nucleotides indicate mismatches.

**Table 2.** Correlation coefficients of days to maturity and flowering between pairs of trial years

	DtM2021	DtM2022	DtM2023	DtF2022	DtF2023
DtM2021					
DtM2022	0.668***				
DtM2023	0.648**	0.870***			
DtF2022	0.634***	0.820***	0.658***		
DtF2023	0.501*	0.563***	0.690***	0.597***	

* *P* < 0.05; ** *P* < 0.01; *** *P* < 0.001.

**Table 3.** GWAS scores of days to maturity (DtM) and days to flowering (DtF) in the 45.7–50.6-Mb region on Pv01

SNP position on chromosome 1	LD block number*^a^*	Score (–log_10_*P*)*^b^*			
		DtM 2021	DtM 2022	DtM 2023	DtF 2022
45 781 409	12	0.22	0.50	0.69	0.32
46 548 974	12	1.68	2.57	1.03	0.57
46 817 081	–	0.82	1.97	1.24	0.21
47 085 595	13	1.88	2.54	1.52	0.98
47 100 084	13	1.38	2.59	2.26	0.74
47 419 094	–	0.81	3.42	0.70	2.46
47 479 438	14	7.84	11.88	5.09	5.24
47 479 506	14	8.03	12.58	5.08	5.83
47 892 313	–	1.98	8.45	1.09	3.87
50 630 505	15	0.06	0.63	0.06	0.36
50 630 540	15	0.05	0.62	1.52	0.35
50 630 551	15	0.06	0.63	0.01	0.37
50 630 646	15	0.05	0.63	0.03	0.36

*^a^* Linkage disequilibrium (LD) blocks were estimated according to the Dʹ value using the method of Gabriel *et al.* (2002). Sixteen LD blocks were found on Pv01. *^b^* White number on a black background means significant (FDR, *P* = 0.05).

**Table 4.** SNP and SSR marker genotypes of ten cultivars used for genotyping

Maturity group	Cultivar or line	Ave. maturity*^a^* (days)	SSR_​m47470k (bp)	SNP_​47479438	SNP_​47479506	SSR_​m47528k (bp)	SSR_​m47621k (bp)	Insertion_​47644850*^d^*	SSR_​m47782k (bp)	SNP_​47892313	SSR_​m47913k (bp)
Early	Taisho-Kintoki	91.7	303	A	C	256	336	C	191	C	215
	Fukura-Kintoki	90.8	303	NA*^b^*	NA*^c^*	256	336	C	191	C	215
	Akibare	91.5	303	A	C	256	336	C	191	C	215
	Kita-Rosso	94.3	303	A	C	256	336	C	191	C	215
	Tokei-B524	92.0	303	A	C	256	336	C	191	C	215
Middle	Kachidoki	97.5	311	T	G	284	320	–	197	T	201
	Fukumasari	97.2	311	T	G	279	320	–	195	T	201
	Hokkai-Kintoki	99.5	307	T	G	273	320	–	197	T	201
	Fuku-Shirokintoki	97.5	311	T	G	282	320	–	195	T	201
	Fuku-Uzura	98.7	311	T	G	284	320	–	195	T	201

*^a^* Average days to maturity in 2021–2023. *^b^* Not available. The imputed SNP genotype was A. *^c^* Not available. The imputed SNP genotype was C. *^d^* An insertion mutation in exon 1 of *Phvul.001G221100*. The genotypes were determined by Sanger sequencing.

**Table 5.** The effects of the Taisho-Kintoki allele on days to maturity in the GWAS population

Year	Estimated genetic effect (days)*^a^*	R^2^ (%)
2021	–5.6	40.5
2022	–7.3	66.3
2023	–4.8	57.0
Combined three-year data	–5.9	70.0

*^a^* The effect of the Taisho-Kintoki allele on days to maturity.
