# Supplementary material for: Genome-wide association mapping for early maturity in kintoki bean (Phaseolus vulgaris L.)
Source: Breed Sci. 2025 Mar 27;75(2):119–28. doi: 10.1270/jsbbs.24054 (PMC12395197; doi:10.1270/jsbbs.24054)
Supplement: Supplementary file 1 — Supplemental Figures [file 75_119_s1.pdf]

doi: 10.1270/jsbbs.24054

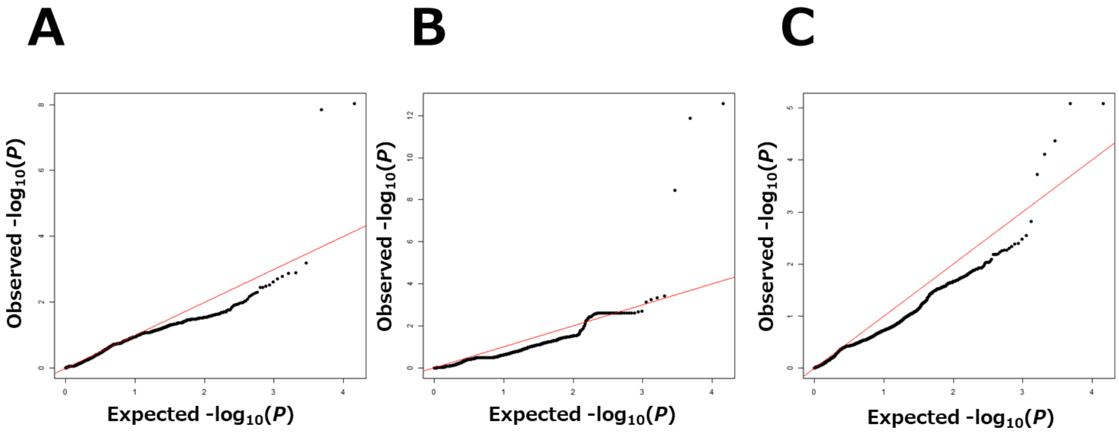

Supplemental Fig. 1. Q–Q plot. Red line shows the 45-degree reference line. (A) Year 2021. (B) Year 2022. (C) Year 2023.

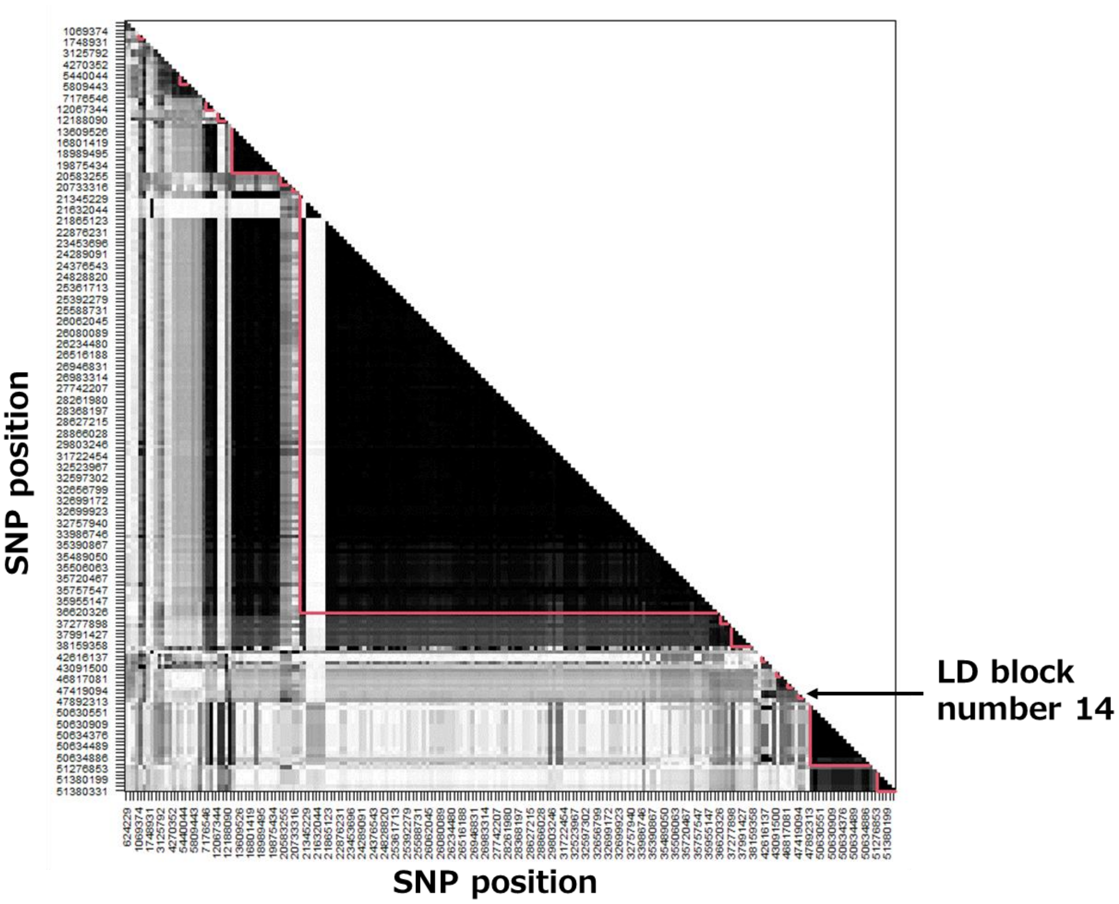

Supplemental Fig. 2. Estimated linkage disequilibrium (LD) blocks according to the D′ value using the method of Gabriel *et al.* (2002). Sixteen LD blocks were found on Pv01. SNP_47479438 and SNP_47479506 belong to LD block 14.

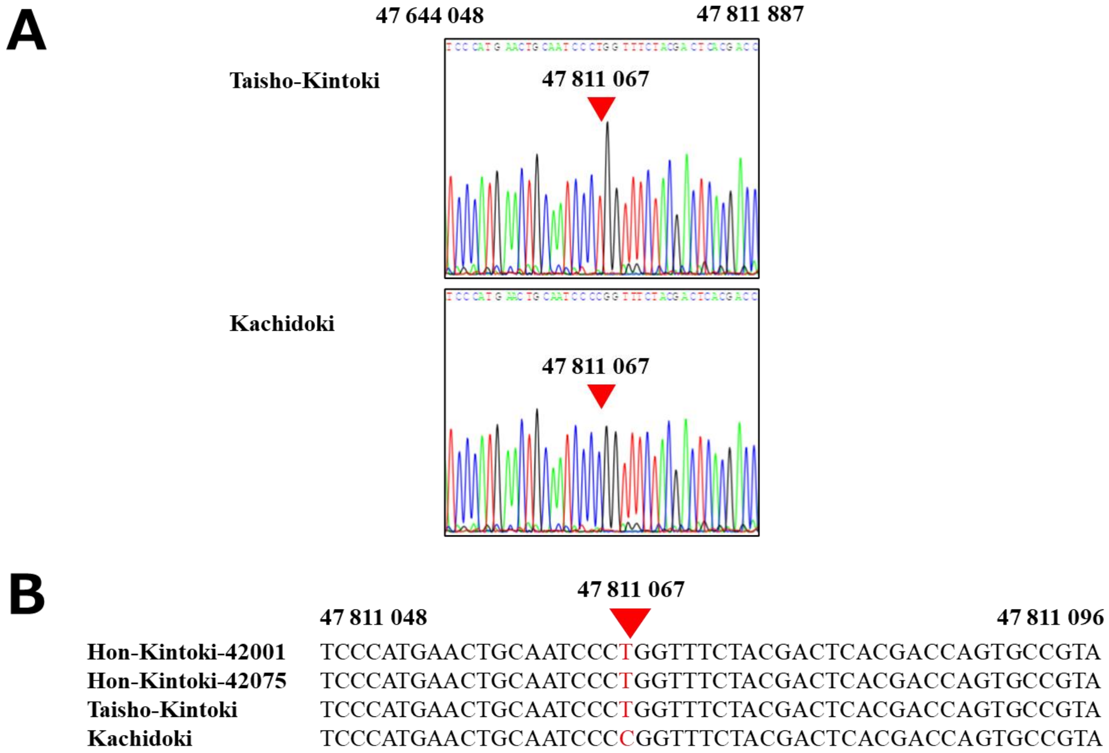

Supplemental Fig. 3. Confirming a non-synonymous SNP in exon 1 of *Phvul.001G223000* by Sanger sequencing. Sanger sequencing used forward primer SEC24-1 (5’-AGA AGA CTT CAA TGC CCT C-3’) and reverse primer SEC24-2 (5’-ATC TTC TGC AGC GAA CAA CAC-3’). (A) Representative sequence chromatograms of Taisho-Kintoki (T, alternative) and Kachidoki (C, reference). (B) Comparison of sequences of the 4 cultivars.
